# Dose-escalated radiotherapy to 82 Gy for prostate cancer following insertion of a peri-rectal hydrogel spacer: 3-year outcomes from a phase II trial

**DOI:** 10.1186/s13014-022-02103-5

**Published:** 2022-07-25

**Authors:** Andrew W. See, Patrick Bowden, Geoffrey Wells, Sree Appu, Nathan Lawrentschuk, Peter Liodakis, Chloe Pandeli, Yolanda Aarons, Lloyd M. L. Smyth, Dean P. McKenzie

**Affiliations:** 1 Icon Cancer Centre, Richmond, Australia; 2 Urology Department, Eastern Health, Box Hill Hospital, Box Hill, Australia; 3 Department of Surgery, Monash University, Melbourne, Australia; 4 Cabrini Health, Malvern, Australia; 5 Department of Urology, Austin Health, Heidelberg, Australia; 6 Division of Cancer Surgery, Peter MacCallum Cancer Centre, Melbourne, Australia; 7 Department of Urology, Royal Melbourne Hospital, Melbourne, Australia; 8 Department of Surgery, University of Melbourne, Melbourne, Australia; 9 EJ Whitten Centre for Prostate Cancer Research, Epworth Healthcare, Melbourne, Australia; 10 North Eastern Urology, Heidelberg, Australia; 11 Icon Institute of Innovation and Research, South Brisbane, Australia; 12 Research Development and Governance Unit, Epworth HealthCare, Richmond, Australia; 13 Department of Health Sciences and Biostatistics, Swinburne University of Technology, Hawthorn, Australia

**Keywords:** Dose-escalation, Hydrogel, Prostate cancer, Radiotherapy, Rectum, Toxicity

## Abstract

**Background:**

Dose-escalation to above 80 Gy during external beam radiotherapy for localised prostate cancer leads to improved oncological outcomes but also substantially increased rectal toxicity. The aim of this study was to demonstrate the safety and efficacy of escalating the dose to 82 Gy following insertion of a peri-rectal hydrogel spacer (HS) prior to radiotherapy.

**Methods:**

This was a single arm, open-label, prospective study of men with localised prostate cancer who were prescribed a course of intensity modulated radiotherapy escalated to 82 Gy in 2 Gy fractions following insertion of the SpaceOAR™ HS (Boston Scientific, Marlborough, MA). Patients were prescribed a standard course of 78 Gy in 2 Gy fractions where rectal dose constraints could not be met for the 82 Gy plan. The co-primary endpoints were the rate of grade 3 gastrointestinal (GI) and genitourinary (GU) adverse events (CTCAE, v4), and patient-reported quality of life (QoL) (EORTC QLQ-C30 and PR25 modules), up to 37.5 months post-treatment.

**Results:**

Seventy patients received treatment on the study, with 64 (91.4%) receiving an 82 Gy treatment course. The median follow-up time post-treatment was 37.4 months. The rate of radiotherapy-related grade 3 GI and GU adverse events was 0% and 2.9%, respectively. There were 2 (2.9%) grade 3 adverse events related to insertion of the HS. Only small and transient declines in QoL were observed; there was no clinically or statistically significant decline in QoL beyond 13.5 months and up to 37.5 months post-treatment, compared to baseline. No late RTOG-defined grade ≥ 2 GI toxicity was observed, with no GI toxicity observed in any patient at 37.5 months post-treatment. Nine (12.9%) patients met criteria for biochemical failure within the follow-up period.

**Conclusions:**

Dose-escalation to 82 Gy, facilitated by use of a hydrogel spacer, is safe and feasible, with minimal toxicity up to 37.5 months post-treatment when compared to rates of rectal toxicity in previous dose-escalation trials up to 80 Gy. Trials with longer follow-up of oncological and functional outcomes are required to robustly demonstrate a sustained widening of the therapeutic window.

*Trial registration* Australian New Zealand Clinical Trials Registry, ACTRN12621000056897, 22/01/2021. Retrospectively registered.

## Background

Dose-escalated radiotherapy up to 78 Gy with conventional fractionation (1.8–2 Gy/fraction), and more recently, moderately hypofractionated radiotherapy (2.5–4 Gy/fraction) are the recommended forms of external beam radiotherapy (EBRT) for low to intermediate risk localised prostate cancer (PCa) [1]. Several randomised trials have demonstrated that dose-escalation reduces biochemical, local and distant failure compared to total doses less than 74 Gy [2–4]. Additional dose-escalation to 80 Gy and above can further reduce the rate of biochemical failure and distant metastases [5, 6] and may improve overall survival for men with high-risk disease [7].

Although improving oncological outcomes, dose-escalated EBRT also increases the rate of late gastrointestinal (GI) and genitourinary (GU) toxicity [2], even when intensity modulated radiotherapy (IMRT) is used [4]. A large randomised trial of EBRT for localised PCa found that rates of grade 2 or higher GI and GU toxicity were 12% and 21%, respectively for the dose-escalated 79.2 Gy arm, versus 7% and 15%, respectively, for the 70.2 Gy arm [4].

The rectum is the dose-limiting organ at risk during dose-escalated EBRT to the prostate [8]. To mitigate the increased risk of rectal toxicity, several methods of increasing the physical separation of the prostate and rectum have been investigated. These strategies include the implantation of a bio-degradable balloon [9] or injectable spacers composed of hyaluronic acid [10] or hydrogel [11, 12], which have been shown to reduce rectal dose [13, 14].

A recent systematic review and meta-analysis found a 66% reduction in the volume of the rectum receiving 70 Gy or more, a 77% reduction in the risk of late grade 2 or higher rectal toxicity and better long-term bowel-related quality of life (QoL) in men with peri-rectal hydrogel spacers implanted prior to dose-escalated prostate radiotherapy [15]. However, there is very limited evidence for the efficacy of peri-rectal hydrogel spacers when the dose is escalated above 80 Gy, with only one retrospective study presenting data following a treatment regimen of 81 Gy in 1.8 Gy fractions [16].

The primary aim of this trial was to evaluate adverse events and QoL up to three years following EBRT to the prostate when escalated to 82 Gy in 2 Gy fractions, following insertion of a hydrogel spacer to minimise rectal dose.

## Methods

### Setting and patients

This is a single centre, single arm, prospective phase II cohort study of dose-escalated IMRT for men with localised PCa following insertion of the SpaceOAR™ hydrogel spacer (HS) (Boston Scientific, Marlborough, MA). The study was approved by the Epworth HealthCare (611-13) and Monash Health (RES-19-0000-167E) human research ethics committees, respectively. All patients provided written informed consent.

Eligible patients were men with pathologically confirmed clinical stage T1 to T3 adenocarcinoma with no evidence of locoregional or distant metastatic disease. Clinical disease stage was determined via digital rectal examination and pre-biopsy magnetic resonance imaging (MRI). Neo-adjuvant or concurrent androgen deprivation therapy (ADT) was permitted at clinician discretion, with the duration of ADT being 6 months and 18 months for patients with intermediate-risk and high-risk disease, respectively. Patients receiving radiotherapy to the pelvic nodes, or who had active inflammatory bowel disease, an active bleeding disorder, or any other malignancy (either active or within 5 years prior to enrolment) except for non-melanoma skin cancer, were excluded from the study.

### Procedures

A baseline computed tomography (CT) scan was performed prior to insertion of the HS. Patients were scanned in a supine position and were required to have a full bladder and empty bowel.

The insertion of the HS took place via a transperineal route under brief general anaesthesia and with antibiotic prophylaxis. The retro-prostatic space was hydro-dissected and 10 mL of the hydrogel was injected into the peri-rectal space under ultrasound guidance using a technique previously described [17]. Three gold-seed fiducials were also inserted into the prostate gland at the time of the HS insertion to facilitate image-guided radiotherapy, as per the standard of care for this cohort.

Planning CT and MRI scans were conducted a minimum of seven days following HS insertion. The prostate, seminal vesicles, HS and organs at risk were delineated following rigid registration of the CT and MRI datasets in the Eclipse™ (Varian Medical Systems, Palo Alto, CA) treatment planning system. High- (82 Gy) and low-dose (59 Gy) planning target volumes (PTV) were defined as 7 mm isotropic expansions of the prostate and seminal vesicles, respectively, except in the posterior direction where a 5 mm margin was applied. The extent of the rectal contour was 1 cm superior and inferior to the PTV.

A seven-field intensity modulated radiotherapy (IMRT) technique was used for treatment planning. Dose objectives for the prostate included mean dose ≥ 82 Gy (100%) and minimum dose ≥ 77.9 Gy (95% of the prescription dose). Dose constraints for the rectum were volume receiving 78 Gy (V78Gy) = 0%, V75Gy < 10%, V70Gy < 20%, V60Gy < 30%, V50Gy < 50% and V30Gy < 60%. Patients were prescribed and treated to 82 Gy unless dose objectives were not met and in that instance they were treated to 78 Gy.

An additional 78 Gy plan using the pre-insertion CT data was generated for each patient for the purpose of comparison to the 82 Gy post-insertion plan with the HS present. The same treatment planning technique was used for both the pre- and post-insertion plans.

Prior to the delivery of each daily fraction, target position and adequacy of bladder (full) and rectal (empty) preparation was verified using a combination of dual orthogonal 2D planar imaging (daily) or 3D volumetric imaging via cone beam CT (weekly at minimum). Image matching was performed based on the position of the three intra-prostatic gold seed fiducials.

Post-EBRT follow-up with clinical assessment, adverse event scoring, serum prostate-specific antigen (PSA) testing, and administration of patient-reported outcome questionnaires was scheduled for 6 weeks post-treatment, three-monthly for 18 months, then at six-month intervals until the final study visit at 37.5 months following the end of treatment.

### Endpoints

Two primary endpoints were evaluated: (1) the incidence of grade 3 or higher GI and GU toxicity, defined by the Common Terminology Criteria for Adverse Events (v4.0), up to 37.5 months following radiotherapy, and (2) patient-reported changes in disease-specific QoL, measured by the European Organisation for Research and Treatment of Cancer (EORTC) Quality-of-life Questionnaire (QLQ) core (C30) [18] and prostate cancer (PR25) [19] modules. Secondary endpoints were the rate of local and biochemical control, respectively. Biochemical failure was defined as a PSA rise of ≥ 2 ng/mL from the post-EBRT nadir [20] or a rising PSA level and radiological evidence of disease progression.

In addition, radiotherapy-related GI toxicity was retrospectively graded according to Radiation Therapy Oncology Group (RTOG) acute and late toxicity criteria. To complement this, the proportion of post-insertion 82 Gy plans with a normal tissue complication probability (NTCP) for late grade ≥ 2 rectal toxicity or rectal bleeding equal to or lower than the corresponding pre-insertion 78 Gy plan was evaluated. NTCP calculation was performed using a previously validated tool [21] and based on the Lyman-Kutcher-Burman model using model parameters defined by QUANTEC [22].

### Statistical analysis

Proportions are presented as percentages with Clopper-Pearson 95% confidence intervals (CI) [23]. Time to biochemical and local progression was analysed using Kaplan–Meier curves.

Baseline health-related QoL scores for selected QLQ-C30 (global quality of life, physical functioning, constipation, diarrhoea) and QLQ-PR25 (urinary function, bowel function) sub-scales were compared to 7.5-, 13.5-, 19.5-, 25.5-, 31.5- and 37.5-month follow-up time-points using linear regression allowing for clustering by time within patients, employing a robust estimator [24, 25]. The linear effect of time between baseline and 36 months post-treatment was analysed for each sub-scale.

In addition, the trajectory of QoL scores (mean and 95% CI) for each sub-scale was presented graphically at the six time-points from baseline up to 37.5 months post-treatment. Clinically important differences in QoL scores between baseline and each time-point were presented and assessed according to sub-scale specific thresholds for the QLQ-C30 [26]. A 10-point threshold was used for the QLQ-PR25 to determine clinically important changes in QoL from baseline, as has been used previously [27]. All statistical analyses were conducted using Stata version 17 (Stata Corporation, College Station, Texas, 2021). The QLQ-C30 was scored within Stata [28], the QLQ-PR25 was scored using R 4.1 (R Foundation for Statistical Computing, Vienna, Austria, 2021) [29]. A *p* value < 0.05 was considered statistically significant.

## Results

### Demographics

Seventy-one patients were enrolled into the study between November 2013 and September 2016. One patient withdrew from the trial prior to receiving treatment. Baseline characteristics for the cohort of 70 men receiving treatment are shown in Table 1. Sixty-four out of the 70 treated patients (91.4% [95% CI: 82.3–96.8%]) completed an 82 Gy treatment course. Six patients received a total dose of 78 Gy; rectal dose constraints could not be met for the 82 Gy plan for five patients and one patient who was prescribed 82 Gy had dose-limiting urinary toxicity and treatment was halted at 78 Gy. The median (25th to 75th percentile) length of follow-up for the cohort was 37.4 months (36.1–38 months).

**Table 1:** Patient characteristics (n = 70)

*Age at baseline*	
Mean (SD)	73.4 (6.3)
*ISUP Grade Group (n %)*	
1	1 (1.4)
2	21 (30.0)
3	18 (25.7)
4	9 (12.9)
5	21 (30.0)
*Clinical stage*	
T1	17 (24.3)
T2	29 (41.4)
T3	21 (30.0)
*Missing*	3 (4.3)
ADT	
Nil	34 (48.6)
Neo-adjuvant	26 (37.1)
Adjuvant	10 (14.3)
*PSA at enrolment (ng/mL)*	
Median (25th to 75th percentile)	10.2 (6.2–17.1)

Data are presented as no. (%) unless otherwise indicated

*ADT* androgen deprivation therapy, *ISUP* International Society of Urological Pathology, *PSA* prostate specific antigen, *SD* standard deviation

### Adverse events

No grade 3 or higher radiotherapy-related GI adverse events were observed. Two (2.9% [95% CI: 0.35–9.9%]) radiotherapy-related GU grade 3 adverse events were reported; one patient with urinary incontinence at 13.5 months and one patient who had a urinary tract obstruction at 4.5 months, however, the latter had undergone a transurethral resection of the prostate prior to radiotherapy.

Two patients (2.9% [95% CI: 0.35–9.9%]) manifested grade 3 adverse events attributable to the HS. One patient reported mild rectal discomfort approximately 3 weeks following HS insertion with pain increasing intermittently over the course of 4 months. MRI findings were unremarkable and symptoms resolved completely by 6 weeks following radiotherapy. One patient reported rectal pain, increasing following insertion of the HS, and was diagnosed with a rectal ulcer two weeks following radiotherapy. This was managed with a drainage catheter with full resolution of symptoms within 2 months.

### QoL

**Fig. 1:**
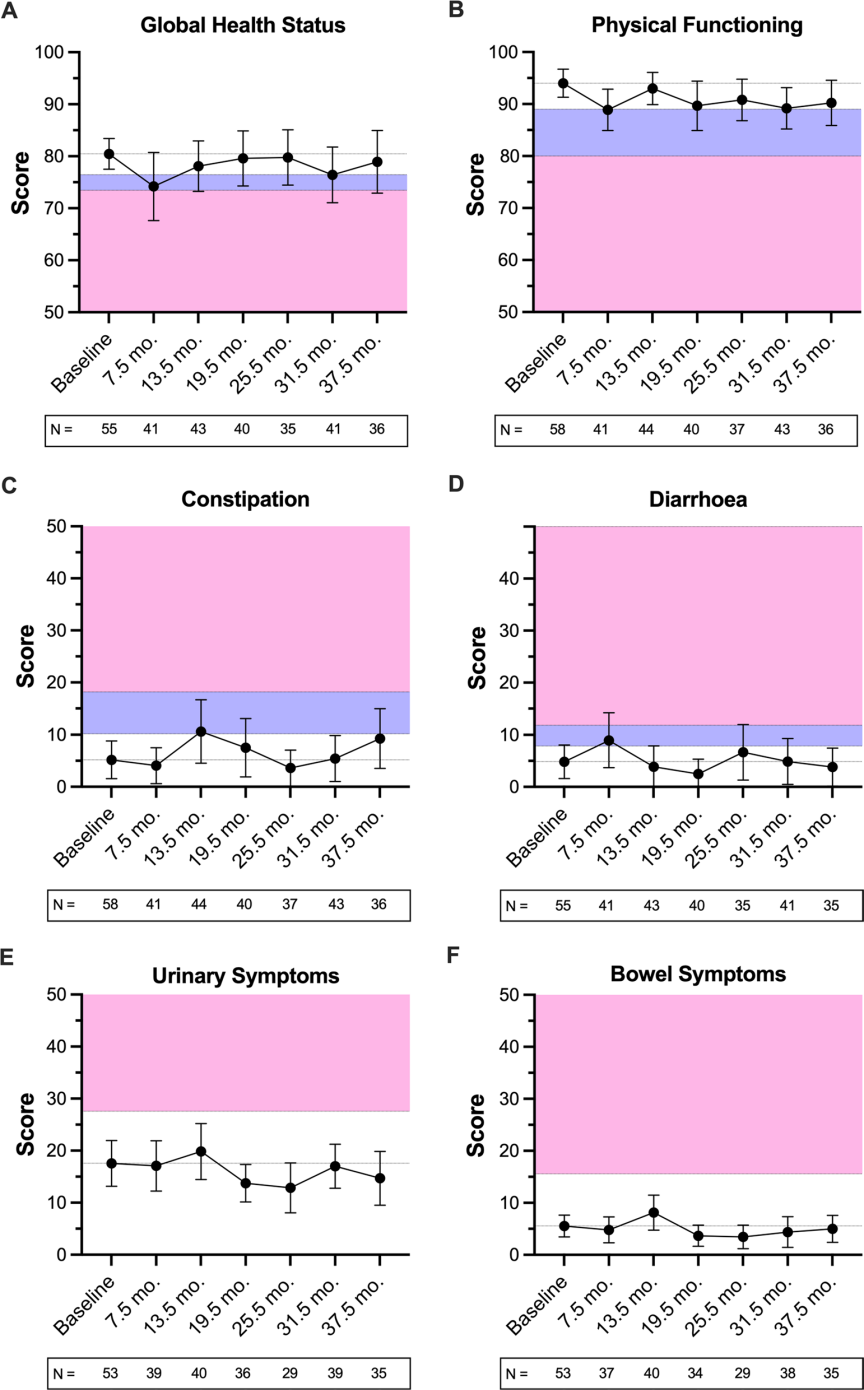
Quality of life trajectory up to 37.5 months following treatment measured by QLQ-C30 (**A**–**D**) and QLQ-PR25 (**E** and **F**) sub-scales. For the QLQ-C30, the blue and pink regions indicate a small and medium clinically important worsening of quality of life, respectively, as previously defined by Cocks et al. 2011 [26]. For the QLQ-PR25, pink regions indicate a minimum 10-point clinically important worsening of quality of life. Small and transient clinically important reductions in global health status (**A**) and physical functioning (**B**) were observed at 7.5 months post-treatment as well as small increases in constipation (**C**) and diarrhoea (**D**) symptom scores at 13.5 and 7.5 months, respectively. No clinically important changes from baseline were observed for overall urinary (**E**) or bowel (**F**) sub-scales

There were no statistically significant changes in bowel related QoL up to 37.5 months following treatment. Constipation and diarrhoea symptom scores transiently exceeded the small clinically important difference threshold (5 and 3 points, respectively) at 13.5 and 7.5 months, respectively, with resolution of QoL scores by the next 6-monthly follow-up time-point for both sub-scales (Fig. 1). No clinically important declines in bowel related QoL beyond 13.5 months post-treatment were observed. Scores for the bowel symptom sub-scale of the QLQ-PR25 did not exceed the clinically important difference threshold within the follow-up period (Fig. 1F).

The mean Physical Functioning score for the cohort was significantly lower than baseline at 7.5 months post-treatment (− 5.1 [95% CI − 9.5 to − 0.8], *p* = 0.022) and 31.5 months (− 4.8 [95% CI − 7.9 to − 1.7], *p* = 0.003). However, only the 7.5-month score exceeded the five-point threshold for a small clinically important difference (Fig. 1B). There was a transient but statistically significant decline in Global Health Status score at 7.5 months (− 6.3 [95% CI − 12.0 to − 0.6], *p* = 0.032) which exceeded the threshold for a small clinically important difference of 4 points (Fig. 1A).

### Observed and predicted rectal toxicity

**Fig. 2:**
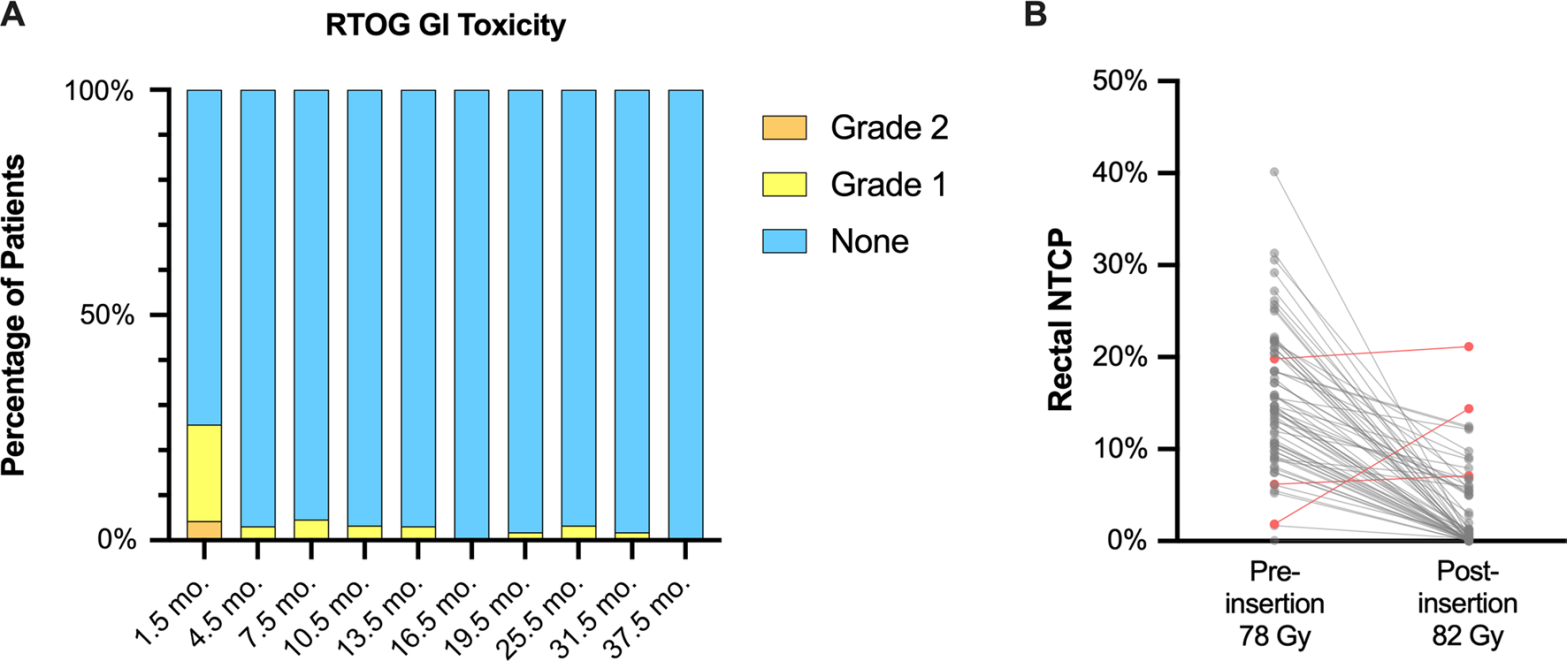
Observed (**A**) and predicted (**B**) GI toxicity up to 37.5 months post-treatment. GI toxicity was most common at 6 weeks post-treatment but resolved almost completely by 4.5 months (**A**). No late grade ≥ 2 toxicity GI toxicity was observed. Sixty-five (95.6% [95% CI: 87.6–99.1%]) of sixty-eight evaluable patients had a reduction in rectal NTCP (late grade ≥ 2 toxicity or rectal bleeding) after insertion of the hydrogel spacer with a prescription dose of 82 Gy compared to a prescription of 78 Gy but with no HS (**B**). The median decrease in NTCP was 11.3%. (95% CI: 9.5–13.0%). The three patients with an increase in rectal NTCP (shown in red) had sub-optimal placement of the spacer at the time of insertion

Rates of acute and late RTOG-defined GI toxicity over the course of the follow-up period are shown in Fig. 2A. GI toxicity was most prevalent at 6 weeks post-treatment, with 15 (21.4% [12.5–32.9%]) and 3 (4.3% [0.9–12.0%]) patients manifesting grade 1 and 2 acute toxicity, respectively, which resolved almost universally across the cohort by 4.5 months post-treatment. Late grade 2 or higher GI toxicity was not observed, and no patient had any GI toxicity at the time of last follow-up.

The NTCP for corresponding pre-insertion 78 Gy and post-insertion 82 Gy plans are shown in Fig. 2B. The NTCP for late grade ≥ 2 rectal toxicity or rectal bleeding was lower for the post-insertion 82 Gy plan for 65 out of 68 (95.6% [95% CI: 87.6–99.1%]) evaluable patients. The increase in NTCP in three cases was attributable to sub-optimal placement of the HS. The median NTCP was 14.3% (95% CI: 12.6–17.2%) and 0.6% (95% CI: 0.3–1.3%), for the pre-insertion 78 Gy and post-insertion 82 Gy plans, respectively, with a median reduction in NTCP between corresponding plans of 11.3% (95% CI: 9.5–13.0%).

### Biochemical and local progression

**Fig. 3:**
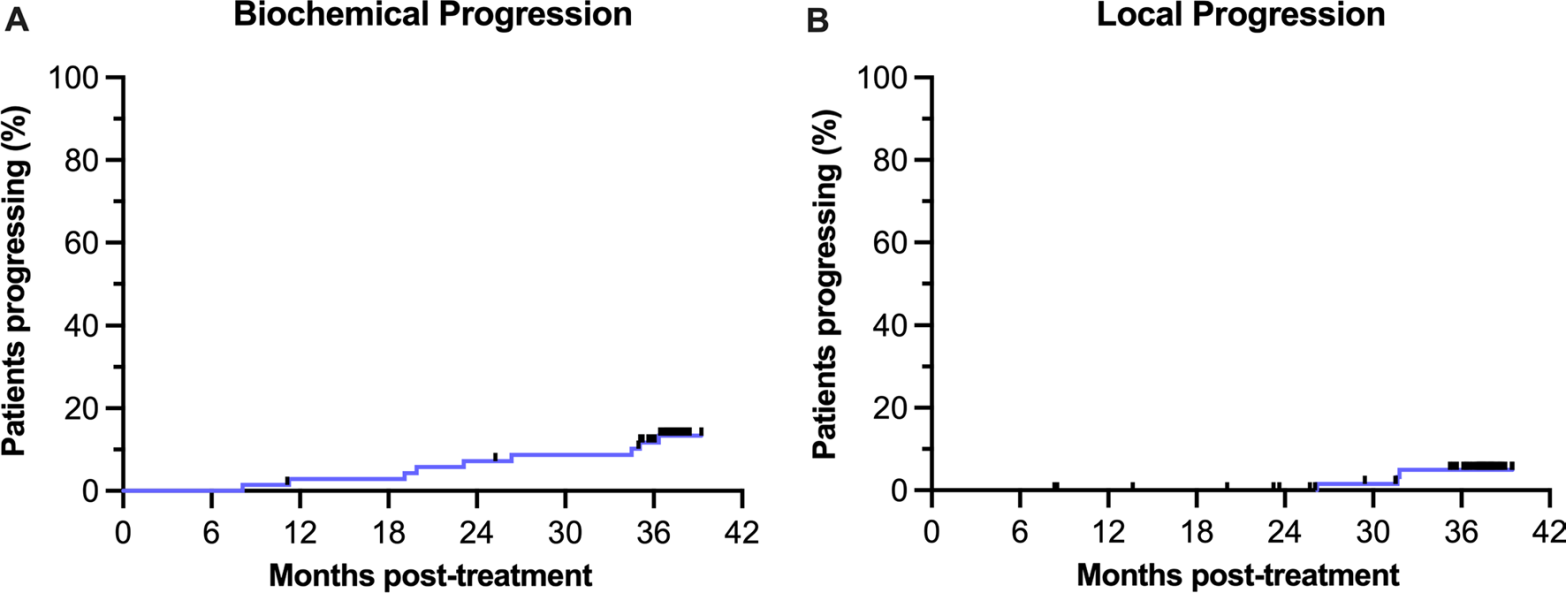
Kaplan–Meier curves of biochemical (**A**) and local (**B**) progression following radiotherapy. Nine (12.9% [95% CI: 6.1–23.0%]) and three (4.3% [95% CI: 0.9–12.0%]) patients had progressed biochemically and locally, respectively, three-years following treatment

Nine (12.9% [95% CI: 6.1–23.0%]) and three (4.3% [95% CI: 0.9–12.0%]) patients met the criteria for biochemical and local progression, respectively, at three years following treatment (Fig. 3). Five of the nine patients who had progressed biochemically had International Society of Urological Pathology (ISUP) grade group ≥ 4 disease at baseline.

## Discussion

To our knowledge, this is the first study to evaluate outcomes of prostate EBRT to an escalated dose of at least 82 Gy (2 Gy per fraction) following insertion of a peri-rectal HS. Principally, this study found that dose escalation to 82 Gy was safe, with minimal radiotherapy-related rectal toxicity and only small, transient increases in bowel-specific QoL symptom scores. Two (2.9%) grade 3 GU adverse events were reported and no late grade 2 or higher GI toxicities were observed. Dose-escalation to 82 Gy was feasible following HS insertion, with a high rate (95.6%) of reduction in rectal NTCP compared to a standard 78 Gy plan with no HS, and a high rate (91.4%) of patients being prescribed and subsequently completing the 82 Gy treatment course.

Previous studies using conventionally fractionated doses ≥ 80 Gy report relatively high rates of clinically significant acute and late GI toxicity in the absence of a rectal spacer. Petrongari et al. [30] (86 Gy in 2 Gy fractions, IMRT) report rates of grade 2 acute GI toxicity and late rectal bleeding of 44% and 18%, respectively, in addition to a 2.5% rate of late grade 3 and 4 GI toxicity. In the GETUG 06 phase III trial, the rate of late grade ≥ 2 GI toxicity in the arm receiving 80 Gy was 19.5% [2]. Even with dose-escalation to only 70–78 Gy, rates of late grade ≥ 2 GI toxicity are reported to be in the order of 5–20% [4, 31, 32], underscoring the efficacy of the HS observed in the current study where no late grade 2 GI toxicities were observed. These findings are consistent with the long-term results of a phase III trial (79.2 Gy in 1.8 Gy fractions, IMRT) which reported 0% versus 5.7% 3-year incidence of late grade ≥ 2 GI toxicity in the HS versus no-HS arms, respectively [33].

The use of a HS during dose-escalated prostate EBRT has previously been shown to result in long-term preservation of bowel-specific QoL. Hamstra et al. [33] reported that patients with a HS had better bowel QoL compared to patients without a HS 3 years following treatment (79.2 Gy in 1.8 Gy fractions, IMRT), with this difference being clinically meaningful. In a retrospective study, Pinkawa et al. [34] found that the group patients treated with a HS had a significantly lower rate of moderate to big problems with bowel urgency and had numerically smaller increases in bowel bother score up to 5 years following treatment (76 or 78 Gy in 2 Gy fractions, IMRT). The current study builds on these observations by demonstrating that preservation of long-term bowel QoL is possible when doses are escalated even further to 82 Gy, if a HS spacer is used.

Two spacer-related grade 3 adverse events were observed in this this study, the nature of which were consistent with previous safety reports for SpaceOAR™. Serious adverse events, including occurrences of rectal ulceration, perforation and fistula have been documented previously [35–37]. A recent trial of carbon ion versus proton therapy for prostate cancer reported that two (2.2%) of 92 patients who had a HS inserted were diagnosed with a grade 3 rectal fistula [38]. In addition the rate of low to moderate grade adverse events related to HS insertion has been reported to be up to 10% [15]. Given that the risk of significant HS-related toxicity is non-zero, the use of a HS should be considered in the context of the projected benefits to QoL and the treatment toxicity profile, for example, where dose-fractionation schemes with equivalent dose in 2 Gy fractions (EQD2) > 80 Gy are used. The potential benefit of a HS for a given patient also depends on individual patient anatomy [39] and clinical risk factors [40], which can be used on a prospective basis to select the patients most suitable for HS insertion prior to treatment.

The rationale for dose-escalation above EQD2 80 Gy is improved biochemical progression free survival, with benefits proportional to dose [5]. For patients with ISUP grade group ≥ 4 disease, dose-escalation to ≥ 80 Gy is also associated with lower risks of biochemical failure, distant metastases and overall survival [7]. In this study, the overall rate of 3-year biochemical failure (Phoenix definition) was 12.9%, and 16.7% in the sub-group of patients who had disease with ISUP grade group ≥ 4. While the length of follow-up in this study is limited, this rate of biochemical failure is likely to be consistent with the reports of other dose-escalation trials, including GETUG 06 which reported a 23.5% 5-year rate of biochemical failure in the 80 Gy arm [2]. Of note, the proportion of patients with ISUP grade group ≥ 4 (42.9%) and clinical stage T3 disease (30%) in this study was higher than the 80 Gy arm of GETUG 06 (6.5% and 13%, respectively). The observation that oncological outcomes following dose-escalation to 82 Gy are comparable with other contemporary dose-escalation trials, but rates of late GI toxicity substantially lower with the use of a HS, is suggestive of a clinically significant widening of the therapeutic ratio. Long-term studies (> 5 year) of dose-escalation above EQD2 80 Gy, whether via conventional or hypofractionated regimens, and with the use of a HS, are therefore warranted.

While this study was conducted in the twilight of the conventional fractionation era for PCa the findings are informative for the application of rectal spacers in the setting of hypofractionation. There has been a paradigm shift towards the use of moderately hypofractionated (2.5–4 Gy/fraction) or ultra-hypofractionated (> 4 Gy/fraction) regimens to reduce patient burden, capitalise on advances in both treatment planning and dose-delivery technologies and exploit the radiobiological characteristics of PCa. Trials of hypofractionated regimens report conflicting results regarding differences in late GI sequelae compared to conventional fractionation [41–43], with some studies reporting higher rates of late GI toxicity following hypofractionation [42, 43]. In the HYPRO trial, higher rates of patient-reported late rectal sequelae (including rectal bleeding, mucous discharge and faecal incontinence) were reported following hypofractionated treatment, with a post-hoc analysis finding a significant local dose–effect relationship between these late GI sequelae and regions of the rectal wall receiving intermediate to high doses [42]. This suggests that the application of a HS in the setting of hypofractionation could be highly effective for the minimisation of late GI toxicity. The use of a HS during prostate radiotherapy has also been shown to reduce intra-fraction prostate motion [44]. This could further reduce the risk of morbidity during the delivery of ultra-hypofractionated regimens, however, long-term clinical outcome data is still maturing [45].

The defined scope of this study was to assess safety and feasibility of dose escalation to 82 Gy with the use of a HS, and therefore the main limitations are a relatively short follow-up for oncological outcomes and the lack of a control arm to assess treatment efficacy. In addition, the rate of QoL completion was relatively poor. However, the objective endpoints of radiotherapy-related adverse events and RTOG-defined acute and late GI toxicity provide robust safety data.

## Conclusions

This study demonstrates for the first time the feasibility and safety of escalating the dose to 82 Gy for intact prostate EBRT when a HS is used. Rates of radiotherapy-related adverse events and toxicity were minimal, with no late grade 2 GI toxicity reported during the 3-year follow-up period and no long-term detrimental impact on QoL. Future studies should prospectively assess whether long-term oncological and functional outcomes of dose-escalation to EQD2 > 80 Gy with use of a HS results in a sustainable widening of the therapeutic ratio for this cohort of patients particularly with the increasing deployment of moderately and ultra-hypofractionated schedules.

## Data Availability

Non-identifiable datasets used and/or analysed during the current study are available from the corresponding author on reasonable request.

## Abbreviations

ADT: Androgen deprivation therapy
CI: Confidence interval
CT: Computed tomography
EBRT: External beam radiation therapy
EORTC: European Organisation for Research and Treatment of Cancer
EQD2: Equivalent dose in 2 Gy fractions
GI: Gastro-intestinal
GU: Genitourinary
HS: Hydrogel spacer
IMRT: Intensity modulated radiation therapy
ISUP: International Society of Urological Pathology
MRI: Magnetic resonance imaging
NTCP: Normal tissue complication probability
PCa: Prostate cancer
PSA: Prostate-specific antigen
PTV: Planning target volume
QoL: Quality of life
QLQ-C30: Quality of life questionnaire core module
QLQ-PR25: Quality of life questionnaire prostate cancer module
QUANTEC: Quantitative Analyses of Normal Tissue Effects in the Clinic
RTOG: Radiation Therapy Oncology Group
SD: Standard deviation
VXX Gy: Proportion of structure receiving a minimum of XX Gy
